# Galaxy workflows for fragment-based virtual screening: a case study on the SARS-CoV-2 main protease

**DOI:** 10.1186/s13321-022-00588-6

**Published:** 2022-04-12

**Authors:** Simon Bray, Tim Dudgeon, Rachael Skyner, Rolf Backofen, Björn Grüning, Frank von Delft

**Affiliations:** 1 Bioinformatics Group, Department of Computer Science, University of Freiburg, Freiburg im Breisgau, Germany; 2 Informatics Matters, Yew Tree Farm, High Street, Charlton on Otmoor, Kidlington, UK; 3 Diamond Light Source Ltd, Harwell Science and Innovation Campus, Didcot, UK; 4 Signalling Research Centres BIOSS and CIBSS, University of Freiburg, Freiburg im Breisgau, Germany; 5 Research Complex at Harwell, Harwell Science and Innovation Campus, Didcot, UK; 6 Structural Genomics Consortium, University of Oxford, Oxford, UK; 7 Department of Biochemistry, University of Johannesburg, Johannesburg, South Africa

**Keywords:** Fragment screening, Workflows, SARS-CoV-2, Computational chemistry

## Abstract

We present several workflows for protein-ligand docking and free energy calculation for use in the workflow management system Galaxy. The workflows are composed of several widely used open-source tools, including rDock and GROMACS, and can be executed on public infrastructure using either Galaxy’s graphical interface or the command line. We demonstrate the utility of the workflows by running a high-throughput virtual screening of around 50000 compounds against the SARS-CoV-2 main protease, a system which has been the subject of intense study in the last year.

**Supplementary Information:**

The online version contains supplementary material available at 10.1186/s13321-022-00588-6.

## Introduction

Computational techniques are commonly used to assess the affinity of small druglike molecules to a biological target molecule, typically a protein, in a process known as virtual screening. Virtual screening is a complex, multi-step process which needs to be performed at a high-throughput level of thousands or millions of input molecules. As a result, workflow management systems such as KNIME [1], CWL [2], Nextflow [3] or Galaxy [4] prove useful to organize analyses, allowing automation and parallelization of commonly used steps and avoiding tedious manual repetition.

In previous work, we published a range of cheminformatics [5] and molecular dynamics tools [6] via the Galaxy platform. Galaxy provides a range of useful features, including a convenient web-based graphical interface, storage of essential metadata such as tool parameters, and easy construction and execution of workflows from component tools, either on the command line or via the graphical interface. Reproducibility of analyses is ensured by the installation of software dependencies using BioConda [7], conda-forge [8], or BioContainers [9]. In addition, we pointed out that using Galaxy provides access to vast public compute infrastructures, including GPU resources for molecular dynamics calculation, such as the denbi and STFC clouds which underpin the European Galaxy server, https://usegalaxy.eu, a distinctive feature which distinguishes Galaxy from other workflow management systems.

Here, we present several new workflows for protein-ligand docking, molecular dynamics and free energy calculation. These workflows are constructed out of simpler building blocks (the component Galaxy tools) and can be either used directly or modified as templates for other similar calculations. We demonstrate the utility of these workflows by running them at high scale on a system which has attracted much recent attention, namely the main protease (Mpro) of the SARS-CoV-2 virus.

**Fig. 1:**
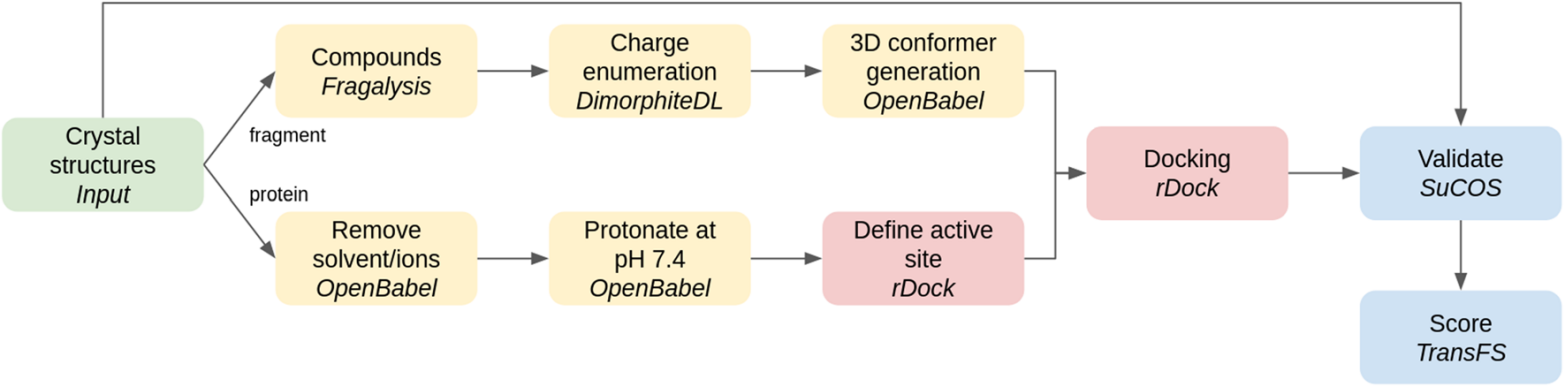
Schematic of the docking and scoring workflow

The main protease of the SARS-CoV-2 virus has been intensively studied since the beginning of the global pandemic, with the first crystal structure released in January 2020 [10]. Subsequent experimental work, involving some of the authors, revealed the crystal structures of Mpro in complex with 96 different fragment structures, including non-covalent hits as well as hits covalently bound to the vital Cys145 residue in the protease binding site [11]. Fragment hits were also found located at the interface between the Mpro dimers. Here we focus our attention on the 22 non-covalent hits bound within the protease active site, excluding two (denoted x1086 and x0887) which bind to other pockets of the protein (the chemical structures of the fragments studied are depicted in Additional file 1: Fig. S1). We use these 22 hits as the basis for generating a list of candidate compounds using the Fragalysis [12] fragment network, a reimplementation of the Fragment Network concept originally developed by Astex Pharmaceuticals [13]. These compounds are then docked using rDock against each of the crystallographic structures from the fragment screen. The resulting docked structures are validated against the original fragment structures using the SuCOS [14] measure and scored using the TransFS [15] deep learning-based method. Based on these scores, the compounds can be ranked and the most promising of them (around 200) used for further free energy calculations. These are performed using the MMGBSA technique, using an ensemble of a total of 5 ns of simulation time per compound. Subsequently we take the 50 top-scoring compounds from the MMGBSA simulations and perform more computationally expensive dcTMD (dissipation-corrected targeted molecular dynamics) [16, 17] calculations, requiring a total of 50 ns of simulation time per compound.

The three workflows themselves (docking and scoring, MMGBSA calculations, and dcTMD calculations) can be flexibly applied to any system, not only Mpro. To facilitate usage by other users in the future, they have been deposited in the Intergalactic Workflow Commission (IWC) [18], a curated repository for Galaxy workflows. To ensure reliability and reproducibility, the workflows are packaged together with tests which are run via continuous integration (CI). If tests are successful and the submission is approved by an IWC review, the submitted workflows are deployed to Dockstore [19] and WorkflowHub [20], two recently developed platforms for sharing scientific workflows. Links for access are provided in Additional file 1: Table S2.

## Methods

Three main workflows have been developed as part of this work: an initial protein-ligand docking and scoring workflow, in which hypothetical protein-ligand structures are generated and ranked; a relatively low-cost free energy calculation workflow, based on the MMGBSA technique, which is run on the most promising of the docked complexes; and a more costly free energy calculation technique, based on the recently published dcTMD method. Subsequent analysis of molecular interactions and plotting of data is performed outside Galaxy. Images of the active site are generated using VMD [21].

### Protein-ligand docking and scoring

The inputs for the docking and scoring workflow consist of a protein structure for docking and a list of candidate compounds. The initial list of candidates is generated with the Fragalysis fragment network API, using the 22 selected fragment hits as inputs to be extended, generating molecules that are close neighbours of the starting molecules in the fragment network.

For those initial candidates, various charge forms between pH 4.4 and 10.4 are enumerated using DimorphiteDL [22]. A single three-dimensional conformer for each of these forms is then produced using OpenBabel [23] as the starting structure for docking. The main task of the workflow, after enumerating charge forms and conformer generation, is to dock each of the enumerated conformers into the binding sites of the fragment crystal structures to generate numerous docking poses, using the open source rDock software [24]. The workflow makes use of the Galaxy’s collection feature to split the initial list of compounds and process the resulting chunks in parallel, essential given the large amount of poses generated. Pocket definition for the docking was achieved by the so-called ‘Frankenstein ligand’ technique of combining atomic coordinates from all fragments into a single hybrid molecule for use as a reference ligand.

**Fig. 2:**
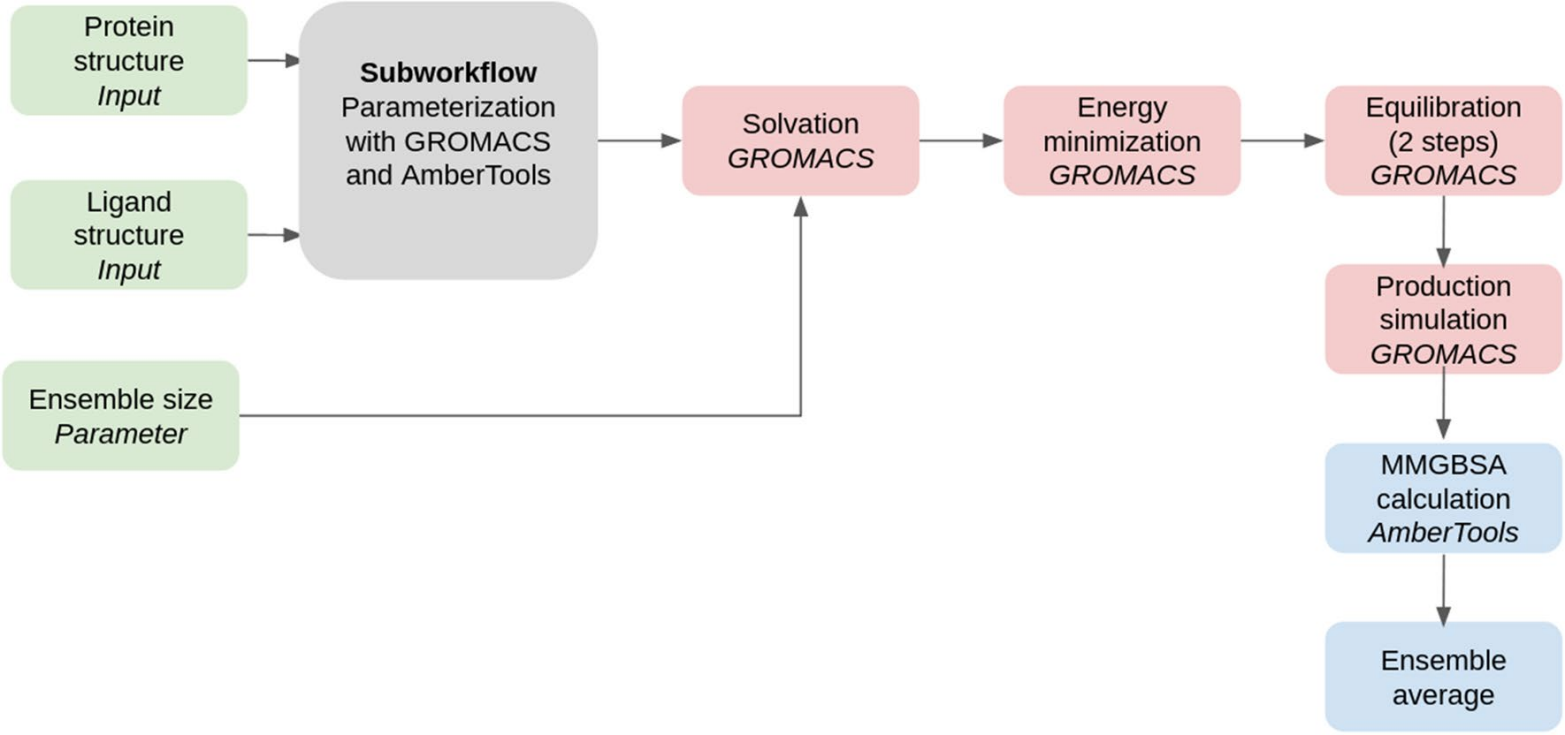
Schematic of the MMGBSA workflow. A modular subworkflow for system parameterization is shared with the dcTMD workflow; see Fig. 4 for details

Docking produced a large number of poses, which were then evaluated using two measures. Firstly, the SuCOS measure is used to assess the overlap between the putative binding position of the compound and each of the experimental fragment crystal structures. The aim is to validate the docked poses and to ensure they share a similar conformation and position to at least one of the experimental crystallographic structures. Secondly, the TransFS tool, based on a deep learning model trained on a variety of molecular interactions, is used to score each of the poses.

A schematic of the workflow is provided in Fig. 1. For our concrete use case, we provide an initial list of 53,787 compounds, which are generated by the Fragalysis fragment network. After charge enumeration and conformer generation, this value is expanded to 219,247, or around 4 conformers per compound. For each of these, 25 docking poses are generated, giving a total of over 5 million poses.

It should be noted that this workflow is run separately for each of the fragment crystal structures, i.e. 22 times, corresponding to a total of over 120 million docking poses. Poses are thus validated against a single fragment during the SuCOS scoring stage. As a result, for each fragment, we obtain a separate list of poses which are ranked only on the basis of their overlap with that single fragment. All poses are also scored using the TransFS tool.

A customizable subworkflow is responsible for filtering the poses based on the assigned scores. Filtering proceeds by selecting the top 5000 compounds for each fragment (around 0.1%) by SuCOS score. As a rule of thumb, a SuCOS score of over 0.5 is acceptable; thus, all poses which differ substantially in conformation and position from the experimental structures are discarded. This subset of poses with high SuCOS scores is then filtered further in one of three ways: (1) selecting all with SuCOS > 0.6 and TransFS > 0.9, (2) selecting all with SuCOS > 0.7 and TransFS > 0.8, (3) for all fragments where these two filtering steps resulted in less than 3 outputs, the top 3 poses based on TransFS scores are selected. By applying this complex filtering, we obtain a range of poses which score highly for both TransFS and SuCOS measures, as well as ensuring a wide chemical diversity of poses with all of the component fragments represented. The filtering is implemented using the sdsort and sdfilter commands which are provided alongside rDock.

A tutorial describing the docking and scoring workflow is available via the Galaxy Training Network [25] at https://bit.ly/31vAZpI.

**Fig. 3:**
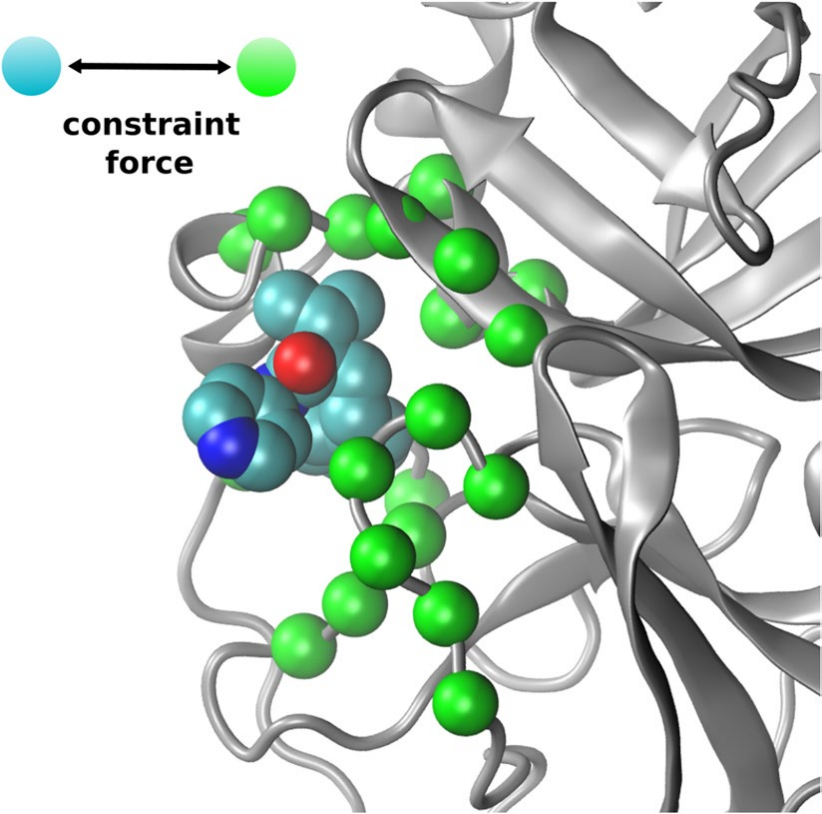
Pull groups for the TMD simulations (image depicts the x0397 structure). Group 1 (cyan) consists of the ligand non-hydrogen atoms. Group 2 (green) consists of a selection of alpha-carbons in the Mpro active site. During the course of the TMD simulation, the two groups are pulled apart by means of a constant constraint force

**Fig. 4:**
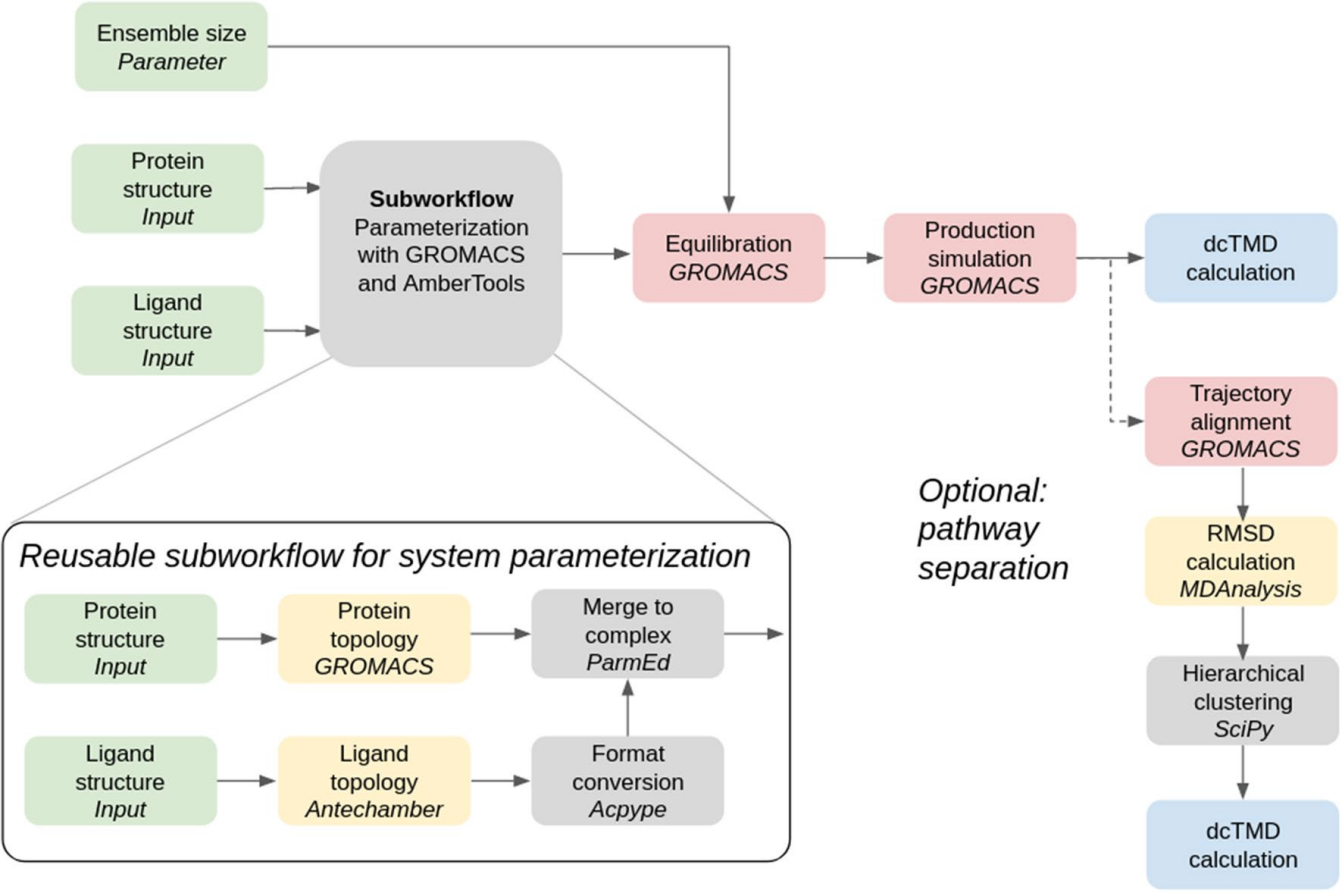
Schematic of the dcTMD workflow

### MMGBSA free energy workflow

The list of compounds obtained after application of the docking and scoring workflow comprises around 210 molecules. To obtain a low-cost assessment of the free energy of binding for each of the poses, we perform MMGBSA calculations, using GROMACS [26] to perform the molecular dynamics simulations and AmberTools [27] for the calculations themselves.

Firstly, a subworkflow for system parameterization is used to prepare the selected ligands for MD simulation. The docked poses are converted from SDF to MOL2 format and parameterized using the GAFF forcefield [28], using tools based on AmberTools and acpype [29]. Meanwhile, the protein structure is parameterized with the AMBER99SB forcefield, using a tool based on GROMACS’s pdb2gmx. Using the tagging system provided by Galaxy, each of the poses is annotated with its respective SuCOS and TransFS value, together with the identity of its parent fragment. These metadata are inherited by datasets produced in subsequent analysis, allowing quick overview of all data for any particular compound.

Solvent (water represented with the TIP3P model) and sodium or chloride counterions are added as required to neutralize the system, before performing energy minimization. The molecular dynamics simulations themselves are performed using GROMACS with a timestep of 1 fs at a temperature of 300 K. 100 ps of equilibration simulations (50 ps under the NVT ensemble followed by 50 ps under the NVT ensemble) are performed with constraints on the protein atoms. The production simulations (length 200 ps) are then performed under the NVT ensemble. For each compound, an ensemble of 20 simulations are performed, taking advantage of Galaxy’s collection functionality to create a list of datasets and apply a tool over the entire list as a single workflow step. The size of the ensemble is configurable as a workflow parameter. The production simulations are then used as a basis for the MMGBSA calculations and a mean across the ensemble is calculated. An schematic of the entire workflow is provided in Fig. 2. It should be noted that the entropic component to the free energy is not included in the calculations, so the values generated represent only the enthalpy of binding.

One of the major reasons to use the Galaxy platform for executing these workflows is that all data, as well as the parameters used for all simulations, are preserved in public Galaxy histories, ensuring full reproducibility. Links to all histories are provided in the Additional file 1.

### dcTMD free energy workflow

As a further demonstration of the capabilities of our tools, and the flexibility of the Galaxy platform which allows them to be combined in numerous different ways, we have designed a third workflow which makes use of the recently published dcTMD free energy technique. The main aim of dcTMD is to provide insight into the kinetics of protein-ligand dissociation; a drug candidate which has a low rate of dissociation from the target protein and thus a high residence time [30] in the binding site will be preferred to a candidate which dissociates quickly. The theoretical background, with comparisons against various common benchmark systems, was provided in two previous publications [16, 17]; the physical basis of the method is described in detail in those two works. The main advantage of the dcTMD method is its provision of free energy and friction profiles for protein-ligand dissociation, with even sampling of the entire reaction coordinate, including areas of high free energy which are infrequently sampled at equilibrium and inherently difficult to study.

**Table 1:** Summary of workflow resource usage

Workflow	CPU time / h	GPU time / h	Data storage / GB	Number of executions	Datasets created
Docking and scoring	3000	1	80	22	6000
MMGBSA	30	2	3	209	893
dcTMD	112	14	6	50	1726

Values for resource usage are approximate and can vary substantially between workflow invocations

The process entails simulation of an ensemble of constraint targeted molecular dynamics (TMD) simulations, in which a constraint pulling force is applied between two atom groups (typically, the ligand and part of the protein) to separate the two groups at constant velocity. The pull groups used for Mpro simulations are depicted in Fig. 3. By applying a weighted average across the ensemble, based on an approximation of the Jarzynski equality [31], free energy and friction profiles for the system at equilibrium can be calculated, despite the fact the ensemble is made up of non-equilibrium simulations.

In order to streamline the process of performing dcTMD calculations, we have developed a complete Galaxy workflow for both simulation and the subsequent calculations. This workflow functions similarly to the MMGBSA workflow, in that it represents the MD ensembles using Galaxy collections, the size of which can be parameterized using a workflow parameter. For dcTMD simulations, an ensemble size of around 100 is recommended [32]; we therefore set ensemble size to 100 for each ligand. MD simulations are performed using GROMACS using a timestep of 1 fs at a temperature of 300 K. 80 ps equilibration is performed under the NPT ensemble with restraints on the protein atoms for each simulation, followed by a 500 ps production TMD simulation under the NPT ensemble without restraints, in which the two pulling groups are separated with a velocity of 1 m/s - in other words, the ligand ends the simulation at 500 pm from its initial position bound in the active site. Pulling simulations are achieved using the PULL code incorporated into GROMACS. As the Mpro binding site is rather shallow, this simulation length is sufficient to sample the entire dissociation pathway. As for the MMGBSA workflow, all data, as well as the parameters used for all simulations, are published in Galaxy histories linked in the Additional file 1.

An essential part of the dcTMD process is pathway separation. One of the core assumptions of the dcTMD protocol is Gaussianity of the work profile of the ensemble, which is acceptable if the ligand takes a uniform path between the bound and unbound state, but breaks down if the ligand is able to take multiple paths out of the binding site. Therefore, it is essential to carry out an analysis to determine whether distinct paths are present in the ensemble. Galaxy tools are also provided to align the TMD trajectories according to the protein atoms and perform hierarchical clustering based on the RMSD between ligand positions. The user then has the option to inspect the clusters manually and to apply the dcTMD calculation again to a subcluster of the ensemble.

A schematic of both the main dcTMD workflow and the optional pathway separation is provided in Fig. 4. Our main aim in calculating the dcTMD free energy profiles is to obtain a value for the maximum free energy reached, which heavily influences the kinetics of dissociation. The position of this barrier on the reaction coordinate is also of interest; by inspecting the free energy and friction profiles generated in combination with the TMD trajectories, links can be made between features of the profiles and events along the unbinding coordinate.

**Fig. 5:**
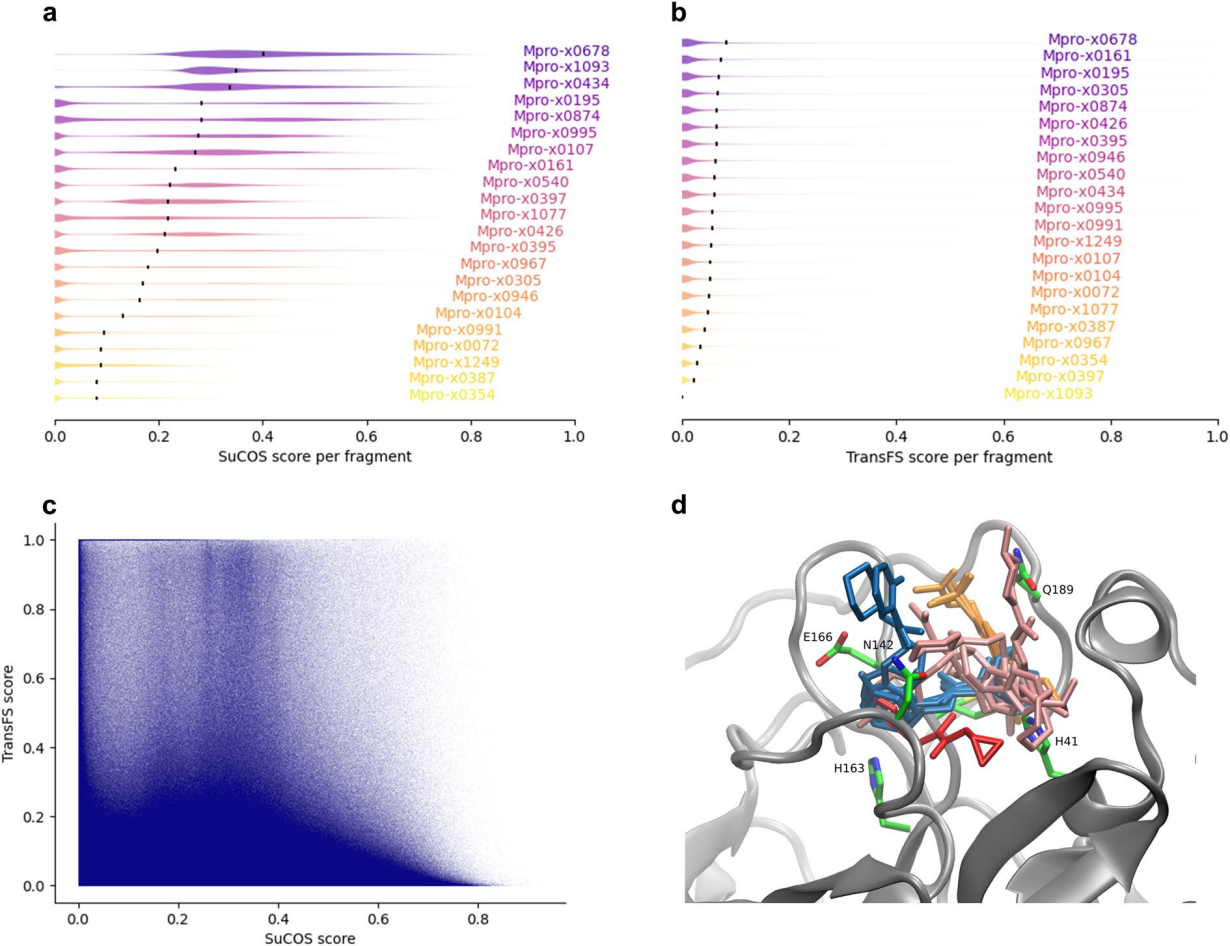
**a** and **b** Distributions of SuCOS and TransFS scores per fragment; the mean values are marked in black. **c** Scatter plot of SuCOS and TransFS scores for all poses. 209 of these are filtered for further screening **d** All fragments superimposed on the protein structure and colored by the main subpocket to which the fragment binds (S1’ red, S1 blue, S2 pink, S3 orange)

### Workflow execution

The workflows detailed here required a high number of executions, particularly in the case of the MMGBSA workflow, which was invoked over 200 times. Galaxy provides a graphical web-based interface for tool and workflow execution, as well as to inspect outputs, but this is of limited use for a project like this one, which requires workflows to be executed several hundred times.

Fortunately, command-line tools are available to automate this process, by providing programmatic access to Galaxy’s API. Workflows are invoked using the command line tool Planemo [33], modifying the input files for each run. This can easily be scaled up using a simple shell script containing a for loop. The Python library BioBlend [34] was also used extensively to move and organize datasets, run individual tools, and restart paused workflows.

Table 1 summarizes execution statistics for each of the workflows. A summary of the number of compounds studied in each step is provided by Table 2.

## Results and discussion

**Table 2:** Number of compounds or poses filtered and studied at each stage

Stage	Fragments	Fragalysis	Docking	MMGBSA	dcTMD
Number of compounds	22	53k	120M	209	49

### Docking

We have assembled three different workflows which can be applied sequentially for virtual screening of a protein. In particular, we have demonstrated the use of these workflows by running them on the SARS-CoV-2 main protease. A key point is that these workflows consist of simple building blocks which can be simply disassembled and recombined to allow different types of analyses and calculations than those covered here. Of the 50000 compounds in our original library, we have identified around 210 docking poses which are scored highly by the TransFS measure, as well as matching the conformations and positions of the component fragments well. For these compounds, we have performed MMGBSA calculations based on ensembles of MD simulations. Additionally, we demonstrate a more computationally intensive dcTMD workflow on a subset of around 50 highly scoring compounds. A summary table is provided in Table 3.

**Fig. 6:**
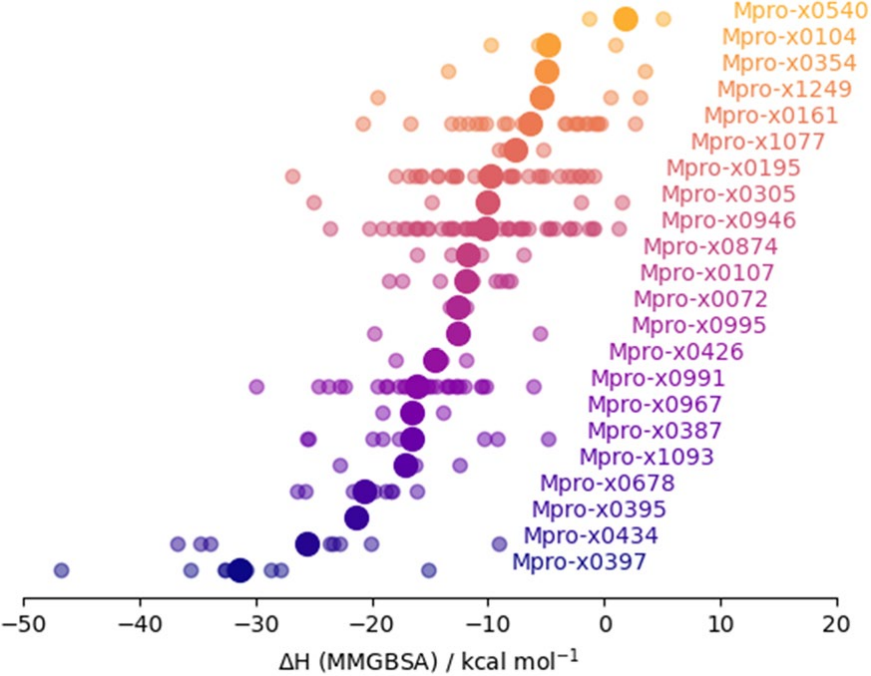
Plot of MMGBSA enthalpies for poses derived from each of the 22 fragments (mean marked by the large circles)

Figure 5a and b shows distributions of TransFS and SuCOS scores per fragment. TransFS scores cluster around a modal value of 0, with a small minority of compounds scoring highly; the 99th percentile lies at 0.61, but the distributions of scores are similar for all the fragments (Additional file 1: Table S1). The single exception is x1093, for which all compounds score effectively 0; the reason for this is difficult to identify, due to the black box nature of the TransFS method, so the TransFS filtering is simply skipped for this fragment. Unlike TransFS, the SuCOS scores are very unevenly distributed, depending on the compound’s parent fragment. It can be observed, for example, that in general smaller fragments such as x0995 score highly, which is unsurprising, as a smaller fragment can fulfil the conditions for overlap more easily. When filtering compounds based on SuCOS score, this should be taken into account, else an unwanted bias towards these smaller fragments is introduced.

Figure 5c demonstrates that the SuCOS and TransFS scores are orthogonal, allowing effective filtering of the compounds on two different measures. While the top right corner of Fig. 5c is relatively sparsely occupied, there are enough compounds present there to select a reasonable number of candidates which score highly on both measures for further study. However, because of a difference between SuCOS score distribution between the different fragments, applying a crude cutoff would ensure certain fragments were heavily overrepresented, while others would remain completely unrepresented. We therefore have developed the more complex filtering workflow described in the Methods section, to ensure all fragments receive some representation in the filtered dataset.

**Fig. 7:**
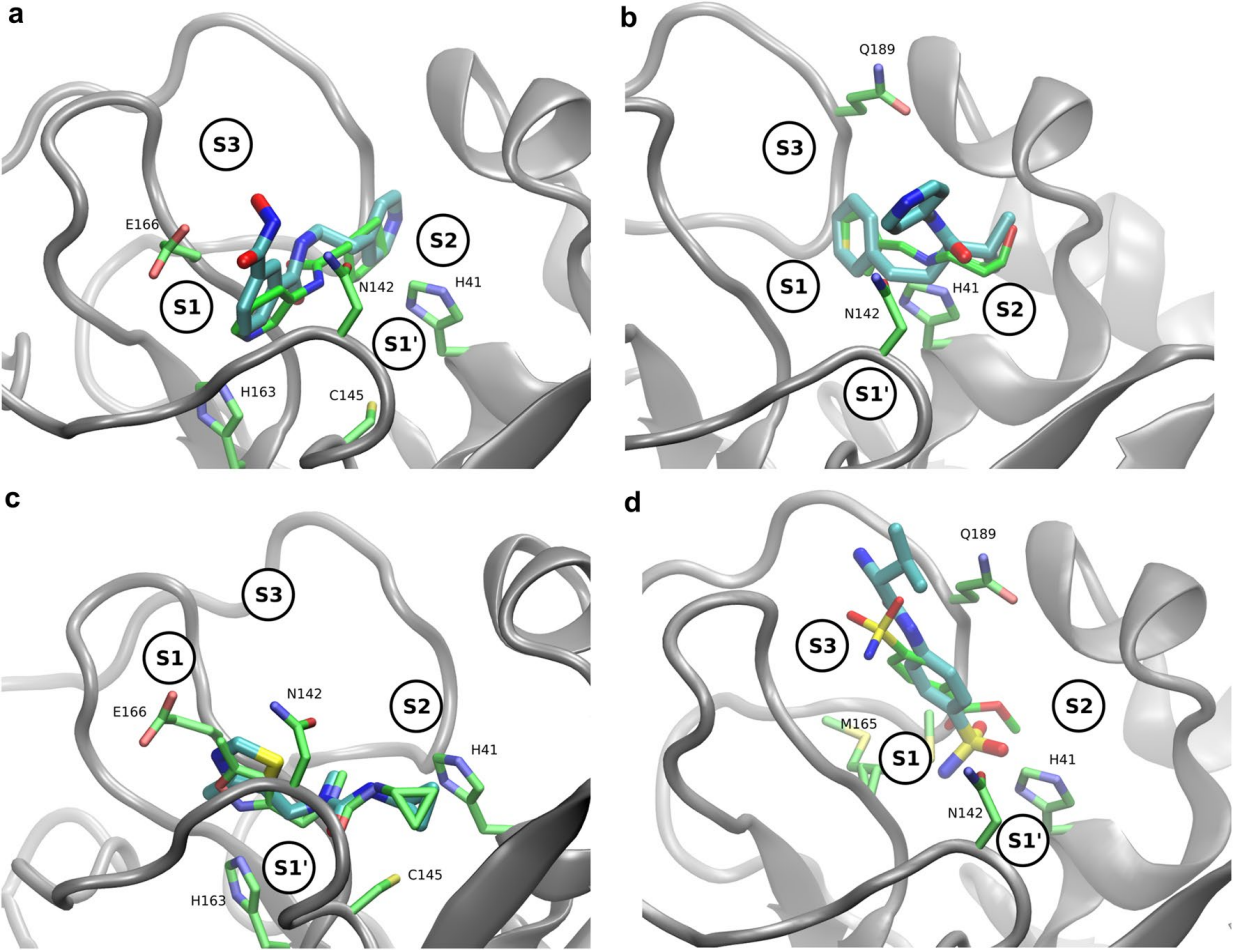
Ligands (cyan) binding in pockets, overlaid on the parent fragments (green): S1’ **a**) (x0397; SuCOS 0.65)), S1 **b** (x0387; SuCOS 0.56), S2 **c** (x0678; SuCOS 0.53) and S3 **d** (x0161; SuCOS 0.60)

**Fig. 8:**
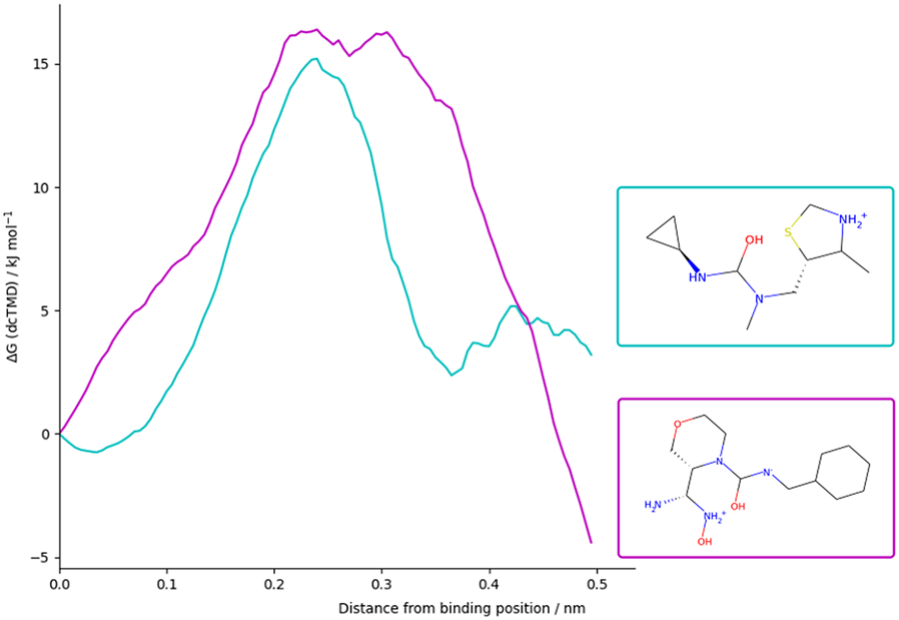
Free energy curves derived from dcTMD calculations for two of the screened compounds

**Fig. 9:**
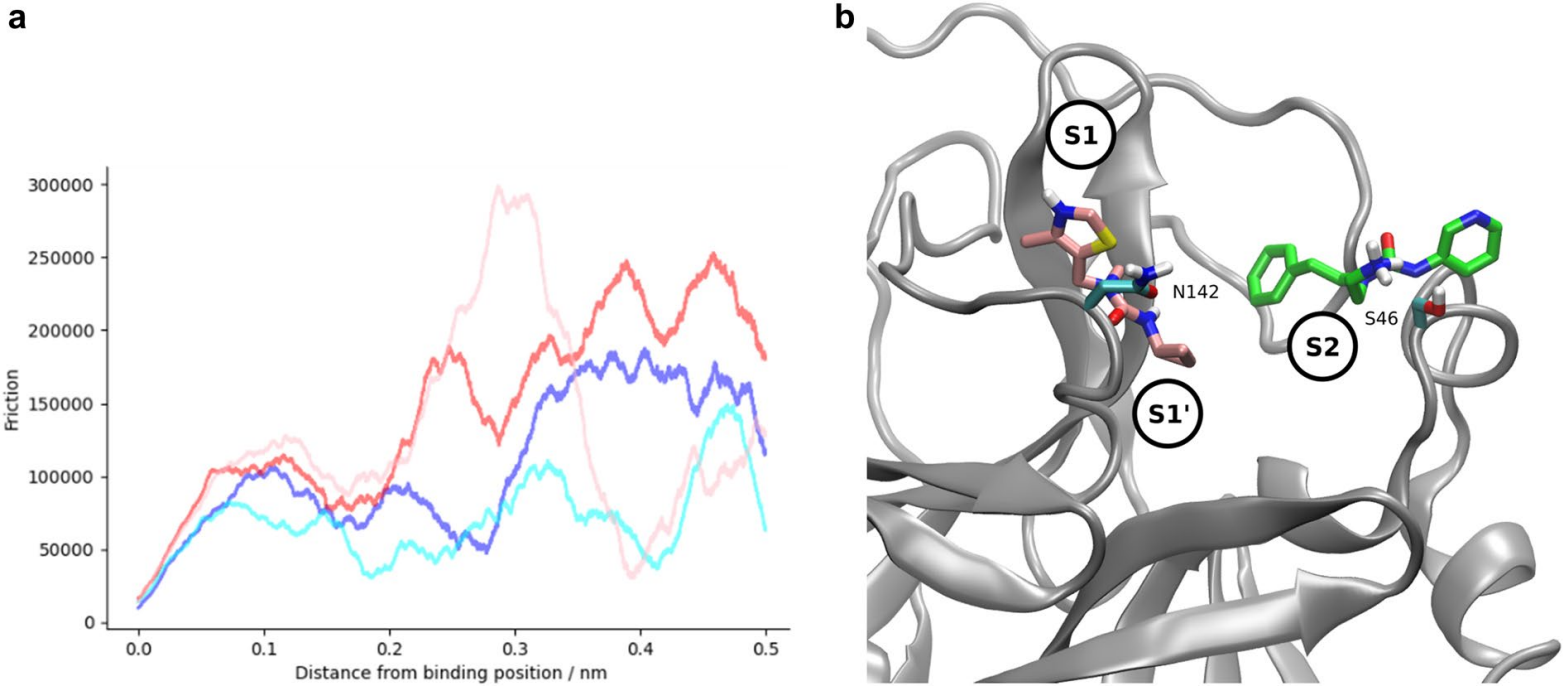
**a** Friction profiles for four selected ligands; the profiles for the ligands binding in subpocket S1/S1’ (red/pink) show a rise starting at 0.2 nm, whereas for those binding in subpocket 2 (blue/cyan), this is absent, with an increase being observable instead at 0.3 nm. **b** Ligands exiting the subpocket S1/S1’ at 0.25 nm from the initial binding position (pink), with Asn142 highlighted, and subpocket S2 at 0.33 nm from the initial binding position (green), with Ser46 highlighted

### MMGBSA

It is interesting to note that the strongest binders, according to the MMGBSA calculations, were those compounds derived from the x0397 fragment (Fig. 6). x0397 is notable as the only fragment which induces a conformational change in the protein; on binding, it displaces the sidechains of the Cys145 and His41 catalytic residues and allows access to an additional subpocket (S1’) to which other fragments cannot bind. Considering the other subpockets, compounds derived from fragments located in both subpockets S1 (e.g. x0434, x0678) and S2 (e.g. x0395, x0387) score highly. On the other hand compounds derived from the three sulfonamide derivatives x0161, x0195 and x0946, which bind in S3, score poorly. Figure 7 depicts four fragments bound to each of the subpockets, together with a derived docking pose superimposed.

**Fig. 10:**
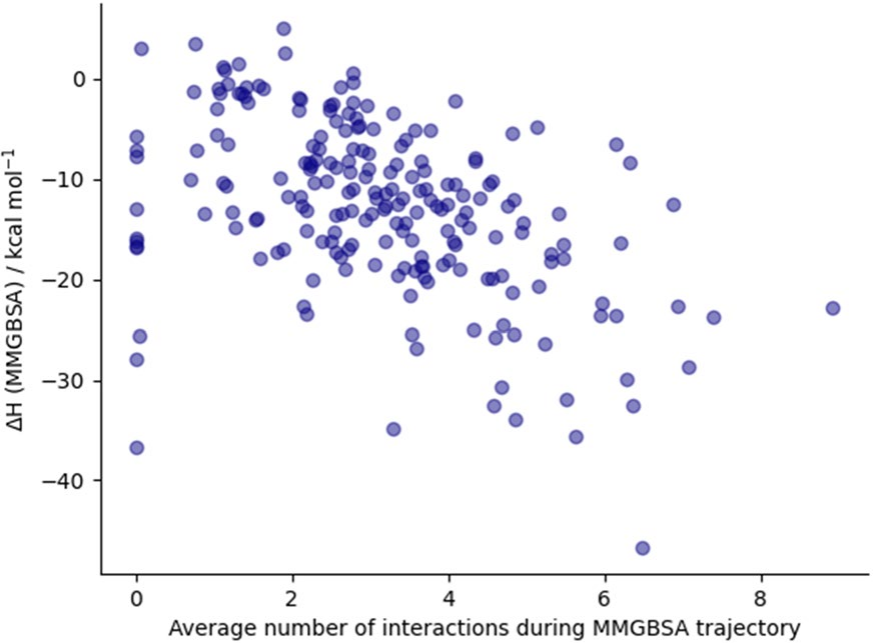
The average number of interactions observed and the free energy as calculated by MMGBSA are correlated ($R^2$ = − 0.46). The weakness of the relationship reflects the high variation in the strength and importance of interactions

**Fig. 11:**
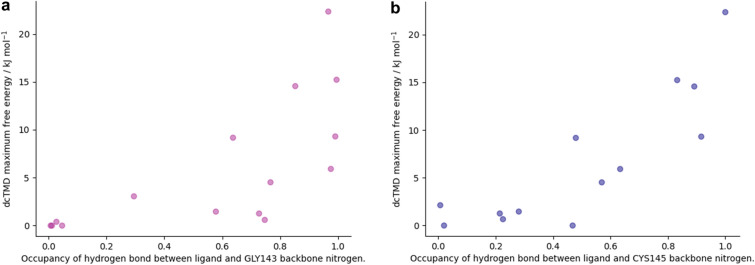
Maximum dcTMD free energy scores for compounds which display hydrogen bonding with the peptide backbone at residues Gly143 ($R^2 = 0.69$) and Cys145 ($R^2 = 0.85$)

Inspection of hydrogen bonds formed during the MD simulations reveals a range of different interactions formed and a wide variation over the set of fragments, as expected. For example, fragment x0678 contains a pyridine group which forms a hydrogen bond with the side chain of His163, buried within subpocket S1. This bond is inherited by several of the compounds derived from x0678. Alternatively, for others of the compounds, the pyridine ring of x0678 is replaced with a hydroxyl or oxime group, which can then form a hydrogen bond with the side chain of Glu166, although the bond does not exist for the fragment itself. Glu166 is also able to form hydrogen bonds with some compounds from its main chain amide group, reflecting its key position at the entrance to subpocket S1.

As it provides access to S1’, x0397 is also the only fragment which enables significant hydrogen bonding with the catalytic cysteine residue.

### dcTMD

Various information can be extracted from the TMD ensemble. Firstly, free energy profiles can be calculated, depicting the free energy of the system relative to the bound state at different points on the pulling coordinate. Friction profiles can also be calculated, depicting the friction present in the system over a particular point in the reaction coordinate. A classic protein-ligand dissociation free energy profile depicts a peak between the bound and unbound state, with the unbound state generally higher in free energy than the bound state (for example, Fig. 8). The height of the peak is of particular interest, as it represents the kinetic barrier to dissociation (Table 3). Secondarily, the position of the peak, or any other features in the free energy or friction profiles, can provide insight into the dissociation pathway, when considered together with manual inspection of the TMD trajectories.

For all of the ligands examined, it appears there is only a single pathway available for ligand dissociation, thus obviating the need to perform the pathway separation step. This is not surprising, given that the binding pocket of Mpro is fairly close to the protein surface.

Inspecting the TMD trajectories, various other interactions become apparent which were not observed in the equilibrium simulations already performed. For the ligands located in the S1 and/or S1’ pockets, such as those derived from fragments x0397 or x0991, an interaction with Asn142 at around 0.25 nm from the binding site can be observed. Asn142 protrudes over the active site, partially covering the entrance to S1 and S1’, where many of the most successful candidate compounds are bound. Therefore, exiting from the binding site entails overcoming a steric clash with the side chain, as well as breaking any transient electrostatic interaction formed with the asparagine side chain. In support of this theory, in the TMD trajectories inspected, the dcTMD free energy peak observed at around 0.3 nm corresponds to the point at which the ligand pushes the side chain aside, having already broken the key molecular interactions, so that no major obstacles now remain to leaving the active site. For fragments exiting from the S2 subpocket, an interaction on the other side of the binding pocket is frequently observed (Fig. 9), with the short helical substructure between amino acids 44 and 50 evident, in particular Ser46, the side chain of which is optimally oriented to face the ligand as it exits the S2 subpocket.

### Interactions

In order to validate the results from the dcTMD and MMGBSA workflows, the interactions between the protein binding site and the docked molecule were systematically examined. This analysis was conducted outside Galaxy using a Python script [35] based on the Open Drug Discovery Toolkit (ODDT) [36]. All hydrogen bonds and hydrophobic interactions between the crystallographic fragments and the binding site were extracted, together with the less frequently occurring salt bridges, $\pi {}$-stacking and $\pi {}$-cation interactions, and halogen bonds. Subsequently, the same script was used to analyse the MMGBSA trajectories produced for each pose, filtering to include only those interactions present in the fragments. By applying the script to one of the equilibrium MD trajectories used for MMGBSA calculation, rather than a static structure, an estimate can be obtained of the occupancy of an interaction over time, rather than simply its presence or absence.

38 interactions were found between the initial 22 fragments and the protein binding site, an average of 1.73 interactions per fragment. By contrast, averaging over the MD trajectories, each compound on average shows 3.13 interactions with the binding site, demonstrating that the method effectively combines multiple fragments to increase the number of protein–ligand interactions. MMGBSA free energies correlate with the number of interactions (Fig. 10), so that considering only the subset of compounds with MMGBSA of less than -20 kcal/mol gives an average of 4.57 interactions.

**Table 3:** Compounds with a maximum dcTMD free energy of over 10 kJ/mol, together with all other calculated scores, and interactions inherited from the component fragments

Index	dcTMD maximum free energy / kJ/mol	Parent (and other component) fragments	Distance of dcTMD maximum from binding site / nm	MMGBSA / kcal/mol	SuCOS	TransFS	Interactions, with occupancy and derived fragment
1	22.41	x0387 (x0434)	0.45	$-$17.74	0.56	0.94	Cys44BO HB 91.5% (x0387)
							Met165 HI 88.5% (x0434)
							Gln189 HI 94.5% (x0434)
							His41 pi stacking 6.5% (x0387)
2	18.4	x0387 (x0434)	0.34	$-$25.51	0.54	0.95	Met165 HI 94% (x0434)
							His41 pi stacking 44% (x0387)
							Gln189 HI 88% (x0434)
3	16.45	x0991 (x0946)	0.24	$-$29.93	0.64	0.96	
4	15.25	x0397	0.24	$-$31.97	0.65	0	Gly143BN HB 100% (x0397)
							Cys145BN HB 83.5% (x0397)
							Thr25 HI 10.5% (x0397)
5	14.57	x0397	0.18	$-$30.74	0.61	0	Gly143BN HB 85.5% (x0397)
							Cys145BN HB 89.5% (x0397)
							Thr25 HI 62.5% (x0397)
6	13.89	x0434	0.38	$-$25.42	0.49	0.65	Glu166BN HB 84.5% (x0434)
							Met165 HI 64% (x0434)
							Gln189 HI 19% (x0434)
7	13.61	x0678	0.73	$-$26.4	0.53	0.94	His163SC HB 14% (x0678)
							Met165 HI 50% (x0678)
							Glu166 HI 90% (x0678)
8	11.96	x0305	0.52	$-$25.07	0.54	0.94	Met165 HI 87.5% (x0305)
							Gln189SC HB 13% (x0305)
9	10.95	x0434	0.43	$-$22.71	0.52	0.68	Gln189 HI 50.5% (x0434)
							Met165 HI 10.5% (x0434)
							Glu166BN HB 3.5% (x0434)
10	10.57	x0434 (x0387)	0.29	$-$34.78	0.52	0.77	Glu166BN HB 77.5% (x0434)
							Met165 HI 61.5% (x0434)
							His163SC HB: 44% (x0434)

The chemical structures of the compounds are depicted in Additional file 1: Fig. S2. *BO* backbone oxygen, *BN* backbone nitrogen, *SC* side chain, *HB* hydrogen bond, *HI* hydrophobic interaction

In addition, a search was also performed for new interactions which do not originate from the crystallographic fragments. This yielded very few results. The most common is a salt bridge between the ligand and Glu166, which is present in 11 molecules with an occupancy > 0.5. Others are even rarer: the second most common interaction not present in the original fragments is a hydrogen bond with the backbone nitrogen of Pro168, for which the maximum occupancy is 0.45; a total of only 7 have an occupancy > 0.1. Considering the chemical diversity of the fragments and their distribution through the binding site, it is not surprising that there is little scope for new interactions to appear, but it helps to confirm that the compounds found successfully replicate the chemistry of the original fragments.

According to Table 3, the majority of the highest-scoring compounds have several high-occupancy interactions inherited from the fragments of which they are composed. In particular, a hydrophobic interaction between Met165 and the ligand is present for almost all the compounds - this interaction is also present for 10 of the 22 original fragments, due to its crucial position at the intersection of the S1 and S2 subpockets. For compounds derived from the x0434 fragment, a hydrophobic interaction with Gln189 and a hydrogen bond with Glu166 also frequently recurs. For compound 3, on the other hand, no interactions can be detected; this is due to the fact that no interactions exist, at least according to the script used, between the parent fragment x0991 and the protein. For the compounds derived from the x0397 fragment, which allows a change in protein conformation and which provided the highest MMGBSA scores, other interactions predominate: hydrogen bonds with Gly143 and Cys145, and to a lesser extent a hydrophobic interaction with Thr25. Both these hydrogen bonds between the ligand and the backbone nitrogen atoms of Gly143 and Cys145 show a particularly strong relationship with the dcTMD free energy score (Fig. 11), and appear only with the x0397 fragment.

The dcTMD scores represent the peak of the free energy profile of dissociation—thus, a high correlation between these interactions and the dcTMD score implies they play an important role in raising the barrier to debinding, where they are present.

## Conclusion

We have presented several new workflows for virtual screening, including protein-ligand docking and scoring, an established free energy technique (MMGBSA) and a more recently developed free energy technique (dcTMD), and demonstrated their use with a study on the main protease of the SARS-CoV-2 virus. These workflows allow us to study a very high number of initial candidate compounds, before narrowing to a smaller selection which we study using more computationally intensive MD techniques. The use of these workflows demonstrates the flexibility of the GROMACS-based MD tools in Galaxy, which can be combined together to create various different types of simulation, including non-equilibrium TMD simulations.

A key motivation for using the Galaxy platform for this kind of study is to enable reproducible, transparent research. Therefore, all datasets are available in the form of published Galaxy histories at https://usegalaxy.eu. Links to the histories are provided in the Additional file 1.

## Supplementary Information

**Additional file 1: Fig. S1.** Fragments used as a basis for the virtual screening. **Table S1.** 99th percentile of TransFS and SuCOS scores per fragment. **Fig. S2.** Top scoring compounds by dcTMD. **Table S2.** Links for accessing the workflows.

## Data Availability

All data is available in the form of published Galaxy histories. Links are provided in Additional file 1.
