# Supplementary material for: Galaxy workflows for fragment-based virtual screening: a case study on the SARS-CoV-2 main protease
Source: J Cheminform. 2022 Apr 12;14:22. doi: 10.1186/s13321-022-00588-6 (PMC9003163; doi:10.1186/s13321-022-00588-6)
Supplement: Supplementary file 1 — Additional file 1: Fig. S1. Fragments used as a basis for the virtual screening. Table S1. 99th percentile of TransFS and SuCOS scores per fragment. Fig. S2. Top scoring compounds by dcTMD. Table S2. Links for accessing the workflows. [file 13321_2022_588_MOESM1_ESM.pdf]

Additional file 1

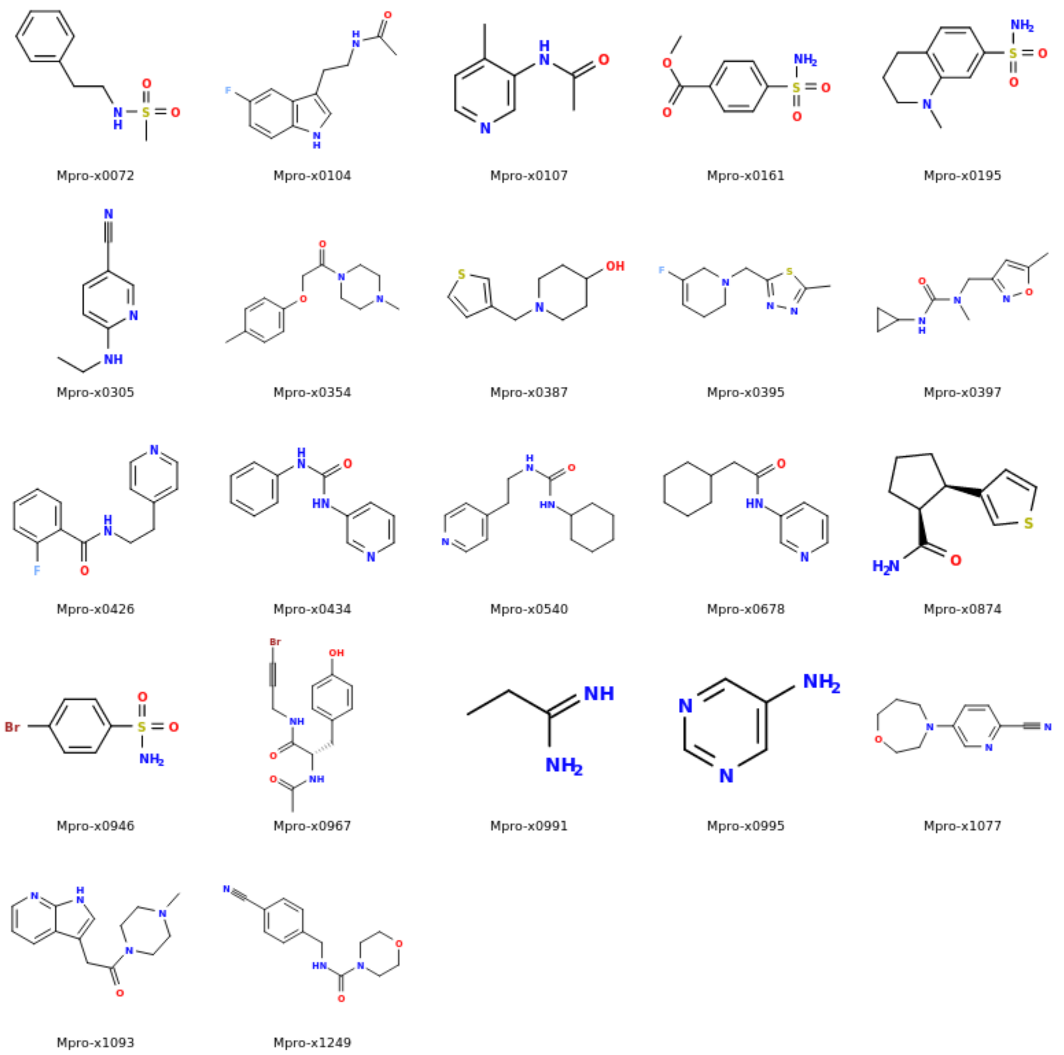

Figure S1. Fragments used as a basis for the virtual screening.

Table S1. 99th percentile of TransFS and SuCOS scores per fragment.

Fragment	SuCOS score	TransFS
Mpro-x0678	0.73	0.89
Mpro-x0161	0.7	0.86
Mpro-x0195	0.69	0.84
Mpro-x0434	0.68	0.81
Mpro-x0874	0.67	0.86
Mpro-x0395	0.66	0.84
Mpro-x0305	0.65	0.86
Mpro-x1077	0.64	0.75
Mpro-x1093	0.63	0
Mpro-x0107	0.55	0.82
Mpro-x0995	0.55	0.86
Mpro-x0967	0.52	0.59
Mpro-x0991	0.49	0.85
Mpro-x0946	0.48	0.82
Mpro-x0540	0.47	0.85
Mpro-x0397	0.46	0.57
Mpro-x0426	0.46	0.86
Mpro-x0104	0.43	0.74
Mpro-x0072	0.39	0.78
Mpro-x0387	0.39	0.73
Mpro-x0354	0.32	1
Mpro-x1249	0.31	0.31

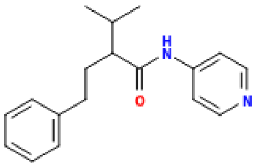

1

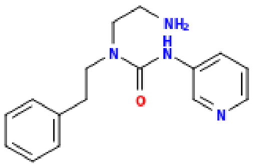

2

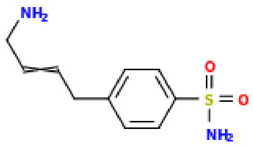

3

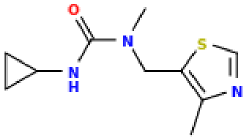

4

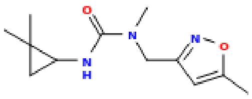

5

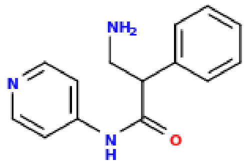

6

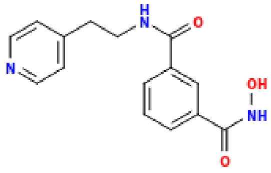

7

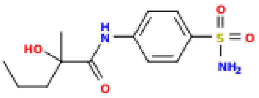

8

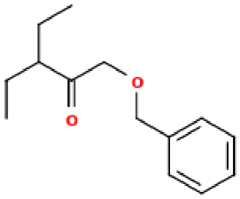

9

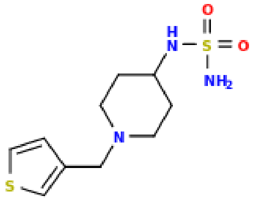

10

Figure S2. Top scoring compounds by dcTMD.

Workflows

The workflows have been published via the Intergalactic Workflow Commission (IWC). From the IWC, they are automatically deployed to Dockstore and the WorkflowHub. In addition, the workflows are publicly available on the European Galaxy server, https://usegalaxy.eu, so users with a Galaxy Europe account can simply log in and start using them.

Table S2. Links for accessing the workflows.

	IWC	Dockstore	WorkflowHub	Galaxy Europe
Docking and scoring	https://github.com/iwc-workflows/fragment-based-docking-scoring/	https://dockstore.org/workflows/github.com/iwc-workflows/fragment-based-docking-scoring/main:main	https://workflowhub.eu/workflows/246	https://usegalaxy.eu/u/sbray/w/fragment-based-virtual-screening-iwc
MMGBSA	https://github.com/iwc-workflows/gromacs-mmgbsa	https://dockstore.org/workflows/github.com/iwc-workflows/gromacs-mmgbsa/main:main	https://workflowhub.eu/workflows/248	https://usegalaxy.eu/u/sbray/w/mmgbsa-calculations-with-gromacs-iwc
dcTMD	https://github.com/iwc-workflows/gromacs-dctmd	https://dockstore.org/workflows/github.com/iwc-workflows/gromacs-dctmd/main:main	https://workflowhub.eu/workflows/249	https://usegalaxy.eu/u/sbray/w/dctmd-calculations-with-gromacs-iwc

List of histories

Docking and scoring:

- https://usegalaxy.eu/u/sbray/h/mpro-x0072
- https://usegalaxy.eu/u/sbray/h/mpro-x0104
- https://usegalaxy.eu/u/sbray/h/mpro-x0107
- https://usegalaxy.eu/u/sbray/h/mpro-x0161
- https://usegalaxy.eu/u/sbray/h/mpro-x0195
- https://usegalaxy.eu/u/sbray/h/mpro-x0305
- https://usegalaxy.eu/u/sbray/h/mpro-x0354
- https://usegalaxy.eu/u/sbray/h/mpro-x0387
- https://usegalaxy.eu/u/sbray/h/mpro-x0395
- https://usegalaxy.eu/u/sbray/h/mpro-x0397

- https://usegalaxy.eu/u/sbray/h/mpro-x0426
- https://usegalaxy.eu/u/sbray/h/mpro-x0434
- https://usegalaxy.eu/u/sbray/h/mpro-x0540
- https://usegalaxy.eu/u/sbray/h/mpro-x0678
- https://usegalaxy.eu/u/sbray/h/mpro-x0874
- https://usegalaxy.eu/u/sbray/h/mpro-x0946
- https://usegalaxy.eu/u/sbray/h/mpro-x0967
- https://usegalaxy.eu/u/sbray/h/mpro-x0991
- https://usegalaxy.eu/u/sbray/h/mpro-x0995
- https://usegalaxy.eu/u/sbray/h/mpro-x1077
- https://usegalaxy.eu/u/sbray/h/mpro-x1093
- https://usegalaxy.eu/u/sbray/h/mpro-x1249

MMGBSA:

- https://usegalaxy.eu/u/sbray/h/mmgbsa-molecule-0
- https://usegalaxy.eu/u/sbray/h/mmgbsa-molecule-1
- https://usegalaxy.eu/u/sbray/h/mmgbsa-molecule-2
- https://usegalaxy.eu/u/sbray/h/mmgbsa-molecule-3
- https://usegalaxy.eu/u/sbray/h/mmgbsa-molecule-4
- https://usegalaxy.eu/u/sbray/h/mmgbsa-molecule-5
- https://usegalaxy.eu/u/sbray/h/mmgbsa-molecule-6
- https://usegalaxy.eu/u/sbray/h/mmgbsa-molecule-7
- https://usegalaxy.eu/u/sbray/h/mmgbsa-molecule-8
- https://usegalaxy.eu/u/sbray/h/mmgbsa-molecule-9
- https://usegalaxy.eu/u/sbray/h/mmgbsa-molecule-10
- https://usegalaxy.eu/u/sbray/h/mmgbsa-molecule-11
- https://usegalaxy.eu/u/sbray/h/mmgbsa-molecule-12
- https://usegalaxy.eu/u/sbray/h/mmgbsa-molecule-13
- https://usegalaxy.eu/u/sbray/h/mmgbsa-molecule-14
- https://usegalaxy.eu/u/sbray/h/mmgbsa-molecule-15
- https://usegalaxy.eu/u/sbray/h/mmgbsa-molecule-16
- https://usegalaxy.eu/u/sbray/h/mmgbsa-molecule-17
- https://usegalaxy.eu/u/sbray/h/mmgbsa-molecule-18
- https://usegalaxy.eu/u/sbray/h/mmgbsa-molecule-19
- https://usegalaxy.eu/u/sbray/h/mmgbsa-molecule-20
- https://usegalaxy.eu/u/sbray/h/mmgbsa-molecule-21
- https://usegalaxy.eu/u/sbray/h/mmgbsa-molecule-22
- https://usegalaxy.eu/u/sbray/h/mmgbsa-molecule-23
- https://usegalaxy.eu/u/sbray/h/mmgbsa-molecule-24
- https://usegalaxy.eu/u/sbray/h/mmgbsa-molecule-25
- https://usegalaxy.eu/u/sbray/h/mmgbsa-molecule-26
- https://usegalaxy.eu/u/sbray/h/mmgbsa-molecule-27

- https://usegalaxy.eu/u/sbray/h/mmgbsa-molecule-28
- https://usegalaxy.eu/u/sbray/h/mmgbsa-molecule-29
- https://usegalaxy.eu/u/sbray/h/mmgbsa-molecule-30
- https://usegalaxy.eu/u/sbray/h/mmgbsa-molecule-31
- https://usegalaxy.eu/u/sbray/h/mmgbsa-molecule-32
- https://usegalaxy.eu/u/sbray/h/mmgbsa-molecule-33
- https://usegalaxy.eu/u/sbray/h/mmgbsa-molecule-34
- https://usegalaxy.eu/u/sbray/h/mmgbsa-molecule-35
- https://usegalaxy.eu/u/sbray/h/mmgbsa-molecule-36
- https://usegalaxy.eu/u/sbray/h/mmgbsa-molecule-37
- https://usegalaxy.eu/u/sbray/h/mmgbsa-molecule-38
- https://usegalaxy.eu/u/sbray/h/mmgbsa-molecule-39
- https://usegalaxy.eu/u/sbray/h/mmgbsa-molecule-40
- https://usegalaxy.eu/u/sbray/h/mmgbsa-molecule-41
- https://usegalaxy.eu/u/sbray/h/mmgbsa-molecule-42
- https://usegalaxy.eu/u/sbray/h/mmgbsa-molecule-43
- https://usegalaxy.eu/u/sbray/h/mmgbsa-molecule-44
- https://usegalaxy.eu/u/sbray/h/mmgbsa-molecule-45
- https://usegalaxy.eu/u/sbray/h/mmgbsa-molecule-46
- https://usegalaxy.eu/u/sbray/h/mmgbsa-molecule-47
- https://usegalaxy.eu/u/sbray/h/mmgbsa-molecule-48
- https://usegalaxy.eu/u/sbray/h/mmgbsa-molecule-49
- https://usegalaxy.eu/u/sbray/h/mmgbsa-molecule-50
- https://usegalaxy.eu/u/sbray/h/mmgbsa-molecule-51
- https://usegalaxy.eu/u/sbray/h/mmgbsa-molecule-52
- https://usegalaxy.eu/u/sbray/h/mmgbsa-molecule-53
- https://usegalaxy.eu/u/sbray/h/mmgbsa-molecule-54
- https://usegalaxy.eu/u/sbray/h/mmgbsa-molecule-55
- https://usegalaxy.eu/u/sbray/h/mmgbsa-molecule-56
- https://usegalaxy.eu/u/sbray/h/mmgbsa-molecule-57
- https://usegalaxy.eu/u/sbray/h/mmgbsa-molecule-58
- https://usegalaxy.eu/u/sbray/h/mmgbsa-molecule-59
- https://usegalaxy.eu/u/sbray/h/mmgbsa-molecule-60
- https://usegalaxy.eu/u/sbray/h/mmgbsa-molecule-61
- https://usegalaxy.eu/u/sbray/h/mmgbsa-molecule-62
- https://usegalaxy.eu/u/sbray/h/mmgbsa-molecule-63
- https://usegalaxy.eu/u/sbray/h/mmgbsa-molecule-64
- https://usegalaxy.eu/u/sbray/h/mmgbsa-molecule-65
- https://usegalaxy.eu/u/sbray/h/mmgbsa-molecule-66
- https://usegalaxy.eu/u/sbray/h/mmgbsa-molecule-67
- https://usegalaxy.eu/u/sbray/h/mmgbsa-molecule-68
- https://usegalaxy.eu/u/sbray/h/mmgbsa-molecule-69
- https://usegalaxy.eu/u/sbray/h/mmgbsa-molecule-70
- https://usegalaxy.eu/u/sbray/h/mmgbsa-molecule-71

- https://usegalaxy.eu/u/sbray/h/mmgbsa-molecule-72
- https://usegalaxy.eu/u/sbray/h/mmgbsa-molecule-73
- https://usegalaxy.eu/u/sbray/h/mmgbsa-molecule-74
- https://usegalaxy.eu/u/sbray/h/mmgbsa-molecule-75
- https://usegalaxy.eu/u/sbray/h/mmgbsa-molecule-76
- https://usegalaxy.eu/u/sbray/h/mmgbsa-molecule-77
- https://usegalaxy.eu/u/sbray/h/mmgbsa-molecule-78
- https://usegalaxy.eu/u/sbray/h/mmgbsa-molecule-79
- https://usegalaxy.eu/u/sbray/h/mmgbsa-molecule-80
- https://usegalaxy.eu/u/sbray/h/mmgbsa-molecule-81
- https://usegalaxy.eu/u/sbray/h/mmgbsa-molecule-82
- https://usegalaxy.eu/u/sbray/h/mmgbsa-molecule-83
- https://usegalaxy.eu/u/sbray/h/mmgbsa-molecule-84
- https://usegalaxy.eu/u/sbray/h/mmgbsa-molecule-85
- https://usegalaxy.eu/u/sbray/h/mmgbsa-molecule-86
- https://usegalaxy.eu/u/sbray/h/mmgbsa-molecule-87
- https://usegalaxy.eu/u/sbray/h/mmgbsa-molecule-88
- https://usegalaxy.eu/u/sbray/h/mmgbsa-molecule-89
- https://usegalaxy.eu/u/sbray/h/mmgbsa-molecule-90
- https://usegalaxy.eu/u/sbray/h/mmgbsa-molecule-91
- https://usegalaxy.eu/u/sbray/h/mmgbsa-molecule-92
- https://usegalaxy.eu/u/sbray/h/mmgbsa-molecule-93
- https://usegalaxy.eu/u/sbray/h/mmgbsa-molecule-94
- https://usegalaxy.eu/u/sbray/h/mmgbsa-molecule-95
- https://usegalaxy.eu/u/sbray/h/mmgbsa-molecule-96
- https://usegalaxy.eu/u/sbray/h/mmgbsa-molecule-97
- https://usegalaxy.eu/u/sbray/h/mmgbsa-molecule-98
- https://usegalaxy.eu/u/sbray/h/mmgbsa-molecule-99
- https://usegalaxy.eu/u/sbray/h/mmgbsa-molecule-100
- https://usegalaxy.eu/u/sbray/h/mmgbsa-molecule-101
- https://usegalaxy.eu/u/sbray/h/mmgbsa-molecule-102
- https://usegalaxy.eu/u/sbray/h/mmgbsa-molecule-103
- https://usegalaxy.eu/u/sbray/h/mmgbsa-molecule-104
- https://usegalaxy.eu/u/sbray/h/mmgbsa-molecule-105
- https://usegalaxy.eu/u/sbray/h/mmgbsa-molecule-106
- https://usegalaxy.eu/u/sbray/h/mmgbsa-molecule-107
- https://usegalaxy.eu/u/sbray/h/mmgbsa-molecule-108
- https://usegalaxy.eu/u/sbray/h/mmgbsa-molecule-109
- https://usegalaxy.eu/u/sbray/h/mmgbsa-molecule-110
- https://usegalaxy.eu/u/sbray/h/mmgbsa-molecule-111
- https://usegalaxy.eu/u/sbray/h/mmgbsa-molecule-112
- https://usegalaxy.eu/u/sbray/h/mmgbsa-molecule-113
- https://usegalaxy.eu/u/sbray/h/mmgbsa-molecule-114
- https://usegalaxy.eu/u/sbray/h/mmgbsa-molecule-115

- https://usegalaxy.eu/u/sbray/h/mmgbsa-molecule-116
- https://usegalaxy.eu/u/sbray/h/mmgbsa-molecule-117
- https://usegalaxy.eu/u/sbray/h/mmgbsa-molecule-118
- https://usegalaxy.eu/u/sbray/h/mmgbsa-molecule-119
- https://usegalaxy.eu/u/sbray/h/mmgbsa-molecule-120
- https://usegalaxy.eu/u/sbray/h/mmgbsa-molecule-121
- https://usegalaxy.eu/u/sbray/h/mmgbsa-molecule-122
- https://usegalaxy.eu/u/sbray/h/mmgbsa-molecule-123
- https://usegalaxy.eu/u/sbray/h/mmgbsa-molecule-124
- https://usegalaxy.eu/u/sbray/h/mmgbsa-molecule-125
- https://usegalaxy.eu/u/sbray/h/mmgbsa-molecule-126
- https://usegalaxy.eu/u/sbray/h/mmgbsa-molecule-127
- https://usegalaxy.eu/u/sbray/h/mmgbsa-molecule-128
- https://usegalaxy.eu/u/sbray/h/mmgbsa-molecule-129
- https://usegalaxy.eu/u/sbray/h/mmgbsa-molecule-130
- https://usegalaxy.eu/u/sbray/h/mmgbsa-molecule-131
- https://usegalaxy.eu/u/sbray/h/mmgbsa-molecule-132
- https://usegalaxy.eu/u/sbray/h/mmgbsa-molecule-133
- https://usegalaxy.eu/u/sbray/h/mmgbsa-molecule-134
- https://usegalaxy.eu/u/sbray/h/mmgbsa-molecule-135
- https://usegalaxy.eu/u/sbray/h/mmgbsa-molecule-136
- https://usegalaxy.eu/u/sbray/h/mmgbsa-molecule-137
- https://usegalaxy.eu/u/sbray/h/mmgbsa-molecule-138
- https://usegalaxy.eu/u/sbray/h/mmgbsa-molecule-139
- https://usegalaxy.eu/u/sbray/h/mmgbsa-molecule-140
- https://usegalaxy.eu/u/sbray/h/mmgbsa-molecule-141
- https://usegalaxy.eu/u/sbray/h/mmgbsa-molecule-142
- https://usegalaxy.eu/u/sbray/h/mmgbsa-molecule-143
- https://usegalaxy.eu/u/sbray/h/mmgbsa-molecule-144
- https://usegalaxy.eu/u/sbray/h/mmgbsa-molecule-145
- https://usegalaxy.eu/u/sbray/h/mmgbsa-molecule-146
- https://usegalaxy.eu/u/sbray/h/mmgbsa-molecule-147
- https://usegalaxy.eu/u/sbray/h/mmgbsa-molecule-148
- https://usegalaxy.eu/u/sbray/h/mmgbsa-molecule-149
- https://usegalaxy.eu/u/sbray/h/mmgbsa-molecule-150
- https://usegalaxy.eu/u/sbray/h/mmgbsa-molecule-151
- https://usegalaxy.eu/u/sbray/h/mmgbsa-molecule-152
- https://usegalaxy.eu/u/sbray/h/mmgbsa-molecule-153
- https://usegalaxy.eu/u/sbray/h/mmgbsa-molecule-154
- https://usegalaxy.eu/u/sbray/h/mmgbsa-molecule-155
- https://usegalaxy.eu/u/sbray/h/mmgbsa-molecule-156
- https://usegalaxy.eu/u/sbray/h/mmgbsa-molecule-157
- https://usegalaxy.eu/u/sbray/h/mmgbsa-molecule-158
- https://usegalaxy.eu/u/sbray/h/mmgbsa-molecule-159

- https://usegalaxy.eu/u/sbray/h/mmgbsa-molecule-160
- https://usegalaxy.eu/u/sbray/h/mmgbsa-molecule-161
- https://usegalaxy.eu/u/sbray/h/mmgbsa-molecule-162
- https://usegalaxy.eu/u/sbray/h/mmgbsa-molecule-163
- https://usegalaxy.eu/u/sbray/h/mmgbsa-molecule-164
- https://usegalaxy.eu/u/sbray/h/mmgbsa-molecule-165
- https://usegalaxy.eu/u/sbray/h/mmgbsa-molecule-166
- https://usegalaxy.eu/u/sbray/h/mmgbsa-molecule-167
- https://usegalaxy.eu/u/sbray/h/mmgbsa-molecule-168
- https://usegalaxy.eu/u/sbray/h/mmgbsa-molecule-169
- https://usegalaxy.eu/u/sbray/h/mmgbsa-molecule-170
- https://usegalaxy.eu/u/sbray/h/mmgbsa-molecule-171
- https://usegalaxy.eu/u/sbray/h/mmgbsa-molecule-172
- https://usegalaxy.eu/u/sbray/h/mmgbsa-molecule-173
- https://usegalaxy.eu/u/sbray/h/mmgbsa-molecule-174
- https://usegalaxy.eu/u/sbray/h/mmgbsa-molecule-175
- https://usegalaxy.eu/u/sbray/h/mmgbsa-molecule-176
- https://usegalaxy.eu/u/sbray/h/mmgbsa-molecule-177
- https://usegalaxy.eu/u/sbray/h/mmgbsa-molecule-178
- https://usegalaxy.eu/u/sbray/h/mmgbsa-molecule-179
- https://usegalaxy.eu/u/sbray/h/mmgbsa-molecule-180
- https://usegalaxy.eu/u/sbray/h/mmgbsa-molecule-181
- https://usegalaxy.eu/u/sbray/h/mmgbsa-molecule-182
- https://usegalaxy.eu/u/sbray/h/mmgbsa-molecule-183
- https://usegalaxy.eu/u/sbray/h/mmgbsa-molecule-184
- https://usegalaxy.eu/u/sbray/h/mmgbsa-molecule-185
- https://usegalaxy.eu/u/sbray/h/mmgbsa-molecule-186
- https://usegalaxy.eu/u/sbray/h/mmgbsa-molecule-187
- https://usegalaxy.eu/u/sbray/h/mmgbsa-molecule-188
- https://usegalaxy.eu/u/sbray/h/mmgbsa-molecule-189
- https://usegalaxy.eu/u/sbray/h/mmgbsa-molecule-190
- https://usegalaxy.eu/u/sbray/h/mmgbsa-molecule-191
- https://usegalaxy.eu/u/sbray/h/mmgbsa-molecule-192
- https://usegalaxy.eu/u/sbray/h/mmgbsa-molecule-193
- https://usegalaxy.eu/u/sbray/h/mmgbsa-molecule-194
- https://usegalaxy.eu/u/sbray/h/mmgbsa-molecule-195
- https://usegalaxy.eu/u/sbray/h/mmgbsa-molecule-196
- https://usegalaxy.eu/u/sbray/h/mmgbsa-molecule-197
- https://usegalaxy.eu/u/sbray/h/mmgbsa-molecule-198
- https://usegalaxy.eu/u/sbray/h/mmgbsa-molecule-199
- https://usegalaxy.eu/u/sbray/h/mmgbsa-molecule-200
- https://usegalaxy.eu/u/sbray/h/mmgbsa-molecule-201
- https://usegalaxy.eu/u/sbray/h/mmgbsa-molecule-202
- https://usegalaxy.eu/u/sbray/h/mmgbsa-molecule-203

- https://usegalaxy.eu/u/sbray/h/mmgbsa-molecule-204
- https://usegalaxy.eu/u/sbray/h/mmgbsa-molecule-205
- https://usegalaxy.eu/u/sbray/h/mmgbsa-molecule-206
- https://usegalaxy.eu/u/sbray/h/mmgbsa-molecule-207
- https://usegalaxy.eu/u/sbray/h/mmgbsa-molecule-208
- https://usegalaxy.eu/u/sbray/h/mmgbsa-molecule-209
- https://usegalaxy.eu/u/sbray/h/mmgbsa-molecule-210
- https://usegalaxy.eu/u/sbray/h/mmgbsa-molecule-211
- https://usegalaxy.eu/u/sbray/h/mmgbsa-molecule--212
- https://usegalaxy.eu/u/sbray/h/mmgbsa-molecule-213
- https://usegalaxy.eu/u/sbray/h/mmgbsa-molecule-214
- https://usegalaxy.eu/u/sbray/h/mmgbsa-molecule-215
- https://usegalaxy.eu/u/sbray/h/mmgbsa-molecule-216
- https://usegalaxy.eu/u/sbray/h/mmgbsa-molecule-217
- https://usegalaxy.eu/u/sbray/h/mmgbsa-molecule-218
- https://usegalaxy.eu/u/sbray/h/mmgbsa-molecule-219
- https://usegalaxy.eu/u/sbray/h/mmgbsa-molecule-220
- https://usegalaxy.eu/u/sbray/h/mmgbsa-molecule-221
- https://usegalaxy.eu/u/sbray/h/mmgbsa-molecule-222
- https://usegalaxy.eu/u/sbray/h/mmgbsa-molecule-223
- https://usegalaxy.eu/u/sbray/h/mmgbsa-molecule-224
- https://usegalaxy.eu/u/sbray/h/mmgbsa-molecule-225
- https://usegalaxy.eu/u/sbray/h/mmgbsa-molecule-226
- https://usegalaxy.eu/u/sbray/h/mmgbsa-molecule-227
- https://usegalaxy.eu/u/sbray/h/mmgbsa-molecule-228
- https://usegalaxy.eu/u/sbray/h/mmgbsa-molecule-229
- https://usegalaxy.eu/u/sbray/h/mmgbsa-molecule-230
- https://usegalaxy.eu/u/sbray/h/mmgbsa-molecule-231
- https://usegalaxy.eu/u/sbray/h/mmgbsa-molecule-232
- https://usegalaxy.eu/u/sbray/h/mmgbsa-molecule-233
- https://usegalaxy.eu/u/sbray/h/mmgbsa-molecule-234
- https://usegalaxy.eu/u/sbray/h/mmgbsa-molecule-235
- https://usegalaxy.eu/u/sbray/h/mmgbsa-molecule-236
- https://usegalaxy.eu/u/sbray/h/mmgbsa-molecule-237
- https://usegalaxy.eu/u/sbray/h/mmgbsa-molecule-238

dcTMD

- https://usegalaxy.eu/u/sbray/h/dctmd-molecule-0
- https://usegalaxy.eu/u/sbray/h/dctmd-molecule-1
- https://usegalaxy.eu/u/sbray/h/dctmd-molecule-2
- https://usegalaxy.eu/u/sbray/h/dctmd-molecule-4
- https://usegalaxy.eu/u/sbray/h/dctmd-molecule-5

- https://usegalaxy.eu/u/sbray/h/dctmd-molecule-6
- https://usegalaxy.eu/u/sbray/h/dctmd-molecule-11
- https://usegalaxy.eu/u/sbray/h/dctmd-molecule-13
- https://usegalaxy.eu/u/sbray/h/dctmd-molecule-18
- https://usegalaxy.eu/u/sbray/h/dctmd-molecule-20
- https://usegalaxy.eu/u/sbray/h/dctmd-molecule-21
- https://usegalaxy.eu/u/sbray/h/dctmd-molecule-23
- https://usegalaxy.eu/u/sbray/h/dctmd-molecule-24
- https://usegalaxy.eu/u/sbray/h/dctmd-molecule-25
- https://usegalaxy.eu/u/sbray/h/dctmd-molecule-26
- https://usegalaxy.eu/u/sbray/h/dctmd-molecule-29
- https://usegalaxy.eu/u/sbray/h/dctmd-molecule-34
- https://usegalaxy.eu/u/sbray/h/dctmd-molecule-37
- https://usegalaxy.eu/u/sbray/h/dctmd-molecule-46
- https://usegalaxy.eu/u/sbray/h/dctmd-molecule-102
- https://usegalaxy.eu/u/sbray/h/dctmd-molecule-106
- https://usegalaxy.eu/u/sbray/h/dctmd-molecule-118
- https://usegalaxy.eu/u/sbray/h/dctmd-molecule-122
- https://usegalaxy.eu/u/sbray/h/dctmd-molecule-123
- https://usegalaxy.eu/u/sbray/h/dctmd-molecule-124
- https://usegalaxy.eu/u/sbray/h/dctmd-molecule-125
- https://usegalaxy.eu/u/sbray/h/dctmd-molecule-126
- https://usegalaxy.eu/u/sbray/h/dctmd-molecule-130
- https://usegalaxy.eu/u/sbray/h/dctmd-molecule-131
- https://usegalaxy.eu/u/sbray/h/dctmd-molecule-137
- https://usegalaxy.eu/u/sbray/h/dctmd-molecule-209
- https://usegalaxy.eu/u/sbray/h/dctmd-molecule-210
- https://usegalaxy.eu/u/sbray/h/dctmd-molecule-211
- https://usegalaxy.eu/u/sbray/h/dctmd-molecule-212
- https://usegalaxy.eu/u/sbray/h/dctmd-molecule-213
- https://usegalaxy.eu/u/sbray/h/dctmd-molecule-215
- https://usegalaxy.eu/u/sbray/h/dctmd-molecule-216
- https://usegalaxy.eu/u/sbray/h/dctmd-molecule-219
- https://usegalaxy.eu/u/sbray/h/dctmd-molecule-220
- https://usegalaxy.eu/u/sbray/h/dctmd-molecule-221
- https://usegalaxy.eu/u/sbray/h/dctmd-molecule-222
- https://usegalaxy.eu/u/sbray/h/dctmd-molecule-223
- https://usegalaxy.eu/u/sbray/h/dctmd-molecule-224
- https://usegalaxy.eu/u/sbray/h/dctmd-molecule-227
- https://usegalaxy.eu/u/sbray/h/dctmd-molecule-229
- https://usegalaxy.eu/u/sbray/h/dctmd-molecule-230
- https://usegalaxy.eu/u/sbray/h/dctmd-molecule-231
- https://usegalaxy.eu/u/sbray/h/dctmd-molecule-233
- https://usegalaxy.eu/u/sbray/h/dctmd-molecule-234

- https://usegalaxy.eu/u/sbray/h/dctmd-molecule-235
- https://usegalaxy.eu/u/sbray/h/dctmd-molecule-236
